# Improving emulsion stability based on ovalbumin-carboxymethyl cellulose complexes with thermal treatment near ovalbumin isoelectric point

**DOI:** 10.1038/s41598-020-60455-y

**Published:** 2020-02-26

**Authors:** Zhenshun Li, Hairui Kuang, Jinchu Yang, Jie Hu, Baomiao Ding, Weiqing Sun, Yangchao Luo

**Affiliations:** 1 College of Life Science, Yangtze University, Jingzhou, 434025 China; 2 Technology Center, China Tobacco Henan Industrial Co., Ltd., Zhengzhou, 450000 Henan China; 3 Department of Nutritional Sciences, University of Connecticut, Storrs, CT 06269 US

**Keywords:** Biophysical chemistry, Biophysics

## Abstract

Ovalbumin (OVA) is an important protein emulsifier. However, it is unstable near the isoelectric point pH, which limits its applications in the food industry. Polysaccharides may be explored to tackle this challenge by improving its pH-dependent instability. In this work, carboxymethyl cellulose (CMC) was used as a model polysaccharide to mix with OVA near its isoelectric point (pH 4.7) with subsequent mild heating at 60 °C for 30 min. The molecular interactions between OVA and CMC were comprehensively studied via a series of characterizations, including turbidity, zeta potential, intrinsic fluorescence, surface hydrophobicity, circular dichroism (CD) spectra and Fourier transform infrared spectroscopy (FTIR). The droplet sizes of the emulsions prepared by OVA-CMC were measured to analyze emulsifying property and stability. The results indicated that free OVA was easily aggregated due to loss of surface charges, while complexing with CMC significantly inhibited OVA aggregation before and after heating owing to the strong electrostatic repulsion. In addition, OVA exposed more hydrophobic clusters after heating, which resulted in the growth of surface hydrophobicity. Altogether, the heated OVA-CMC complexes presented the best emulsifying property and stability. Our study demonstrated that complexing OVA with CMC not only greatly improved its physicochemical properties but also significantly enhanced its functionality as a food-grade emulsifying agent, expanding its applications in the food industry, as development of emulsion-based acidic food products.

## Introduction

Egg ovalbumin (OVA), the main ingredient of egg white protein, is one of the most widely used protein and a kind of important food ingredient. In addition, OVA also possesses important functionalities including emulsifying and foaming stability because it has more than 50% hydrophobic amino acids, similar to other surface-active agents^1,2^. Nevertheless, there are two major limitations for OVA to be widely used in the food industry. First, OVA is known to have poor thermal instability. It was reported that the thermal aggregation temperature of OVA was lower around its isoelectric point than that in other pH conditions, and the optimal thermal stability was in the pH range from 6 to 10^3,4^. In addition, the emulsifying property of OVA was poor near the isoelectric point as shown in our previous work^2^. Meanwhile, as a protein molecule, when the pH is reduced to around its isoelectric point (pH 4.7), OVA carries zero net surface charge and loses its emulsifying capability when used in the emulsion-based acidic food products, such as yogurt and lactic acid beverages^5^.

Carboxymethyl cellulose (CMC), one of the derivatives of cellulose, has been widely used as a stabilizing agent in foods^6^. In addition, CMC is stable with strong negative charges at pH values from 2 to 10, which makes it suitable for various applications in most food products across a wide range of pH conditions. CMC is a commonly used additive to improve the processing properties of products in cosmetics, pharmaceuticals and food industries^7^. Therefore, CMC has been often used as a model polysaccharide to analyze the interactions between proteins and polysaccharides. Geng and co-workers found that the thermal stability of OVA was improved after its glycosylation reaction with CMC^4^. It has also been demonstrated that there is a high affinity between OVA and CMC^8^. A follow-up study revealed that the interfacial activities of heated OVA-CMC complex were greater than that of unheated OVA-CMC complexes^9^. However, the emulsifying property of the complexes after heating was not studied. It was also reported that moderate acid-heat treatment (60 °C for 15 min, pH 3.0) of OVA could increase the thermal stability of OVA-stabilized emulsions^10^. Furthermore, it is well known that charged polysaccharides can reduce the sensitivity of protein solutions against ionic strength and thus inhibit the aggregation of the proteins. From these facts, we hypothesize that heating OVA-CMC complex may enhance the emulsifying property and thus improve the emulsion stability under a wide range of pH conditions, especially at the isoelectric point of OVA. However, there is a lack of comprehensive studies on the emulsifying property under the isoelectric point of OVA after binding with polysaccharides.

In this work, CMC was used to mix with OVA. The OVA-CMC complex solution was heated at 60 °C for 30 min at pH 4.7, which was the thermal unstable state near the isoelectric point of OVA. Moderate heating can expose the hydrophobic groups to the aqueous phase. In addition, CMC will contribute to increasing the electrostatic repulsion among OVA-CMC stabilized emulsion drops. This study is aimed to demonstrate the protective effect of polysaccharides on protein-stabilized emulsions and provide some guidance for tackling the thermal instability of proteins in the preparation of acidic and heated food products.

## Materials and Methods

### Materials

OVA (from egg white, ≥95%, molecular weight 43 kDa) was purchased from Sigma-Aldrich (St. Louis, MO, USA). CMC (molecular weight 90 kDa, degree of substitution 0.7) and 8-anilino-1-naphthalenesulfonate (ANS) were obtained from Aladdin co., ltd (Shanghai, China). Aqueous solutions were prepared by ultrapure water. All of the other reagents were of analytical-grade and were purchased from Aladdin Co., Ltd. (Shanghai, China).

### Preparation of OVA-CMC complex

OVA and CMC were dispersed in deionized water to reach the concentration of 10 g/L, respectively. The solutions were gently stirred for 3 h and were stored overnight at 4 °C to allow complete hydration^11^. The same volume of CMC solution was dropwise added to the magnetic stirring OVA solution with fixing the final OVA concentration as 5 g/L. Then, OVA-CMC complex was prepared with the 1:1 mass ratio of OVA and CMC. Following that, the pH value of the solution was adjusted to 4.7 with hydrochloric acid (0.1 M) solutions. The OVA-CMC solutions were heated at 60 °C for different durations, including 10 min, 20 min, 30 min, 40 min and 50 min. Meanwhile, the unheated OVA-CMC and the same concentration of free OVA solutions were respectively prepared following the same protocol as mentioned above but at room temperature as controls.

### Turbidity and zeta potential measurements of OVA-CMC complexes

The OVA-CMC samples with and without heating were prepared to analyze the turbidities and zeta potentials. Turbidity measurements were acquired at 540 nm by using a Shimadzu UV-1750 spectrophotometer at room temperature (25 °C). Turbidities were calculated as the Eq. (1). Where, A is the absorbance, 1 is the optical path (1 cm) of the cuvette, and 2.303 is a constant.

1 $${\rm{Transmittance}}\,({\rm{T}})\, \% =(10-\frac{2.303A}{1})\times 100 \% $$

The zeta potentials of the OVA-CMC with different heating time were measured by using a Nano ZS 90 (Malvern, Worcestershire, UK). All measurements were carried out at 25 °C with equilibrating for 60 s inside the instrument. The results were reported as averages of three times.

### Surface hydrophobicity

The indexes of surface hydrophobicity (H_0_) of the free OVA and OVA-CMC solutions with different heating duration were analyzed by using ANS as probe to interact with hydrophobic moieties on the surface of OVA to give a fluorescent signal^12^. The OVA-CMC samples were diluted from 0.3125 to 5 g/L, respectively. To study the thermal aggregation of free OVA, OVA samples were diluted to lower concentrations ranging from 0.3125 to 1.25 g/L. Twenty microliters of 8 mM ANS solution (PBS, pH 7.0, 50 mM) were mixed into 4 mL OVA-CMC solutions and kept it in the dark for 15 min. The emission fluorescence intensities were measured by a F4600 fluorescence spectrometer (HITACHI, Tokyo, Japan) with the same emission and excitation slits as 5 nm at a voltage of 500 V, 25 °C. The results were reported as averages of three times. Scatter diagram with maximum fluorescence intensity as ordinate and concentration as abscissa were plotted to make a linear fitting of the graph. H_0_ value is the slope (K) of the line.

### Intrinsic fluorescence measurements

Intrinsic fluorescence intensities of the free OVA, CMC and heated OVA-CMC solutions under different heating time were carried out by using the fluorescence spectrometer from 290 to 450 nm at an excitation wavelength of 280 nm, with the same excitation and emission slit width (5 nm) at 25 °C. The results were reported as averages of three readings.

### Circular dichroism (CD) spectroscopy

The OVA-CMC samples before and after heating at 60 °C for 30 min (abbreviated as H-OVA-CMC) were diluted to 0.2 g/L with ultrapure water and adjusted pH to 4.7. Free unheated OVA sample with the same preparation conditions was used as control. Because OVA was easily aggregated at high concentration at pH 4.7, the heated OVA solution (H-OVA) was prepared at 60 °C for 30 min with the concentration of 0.2 g/L. The CD spectra of the samples were measured from 190 to 250 nm, in a 0.1 cm path length quartz cell with nitrogen gas purging, 1 nm bandwidth and 1 s response time at 25 °C by using a J-1500 spectropolarimeter (JASCO, Tokyo, Japan).

### Fourier transform infrared spectroscopic (FTIR) measurements

The freeze-dried samples of OVA-CMC, H-OVA-CMC, OVA and CMC before and after heating at 60 °C for 30 min were blended with dried potassium bromide at a sample/potassium bromide mass ratio of 1:50 (w/w) and ground into powder, respectively. Then the samples were measured by using a NEXUS 470 spectrophotometer (NICOLET, USA) in the range of 4000–500 cm^−1^ at a resolution of 4 cm^−1^ with air as the background.

### Preparation of the emulsions

The emulsion was prepared by homogenizing the mixtures of soybean oil and the free OVA, OVA-CMC and H-OVA-CMC solutions, respectively. In brief, soybean oil (50 mL) was blended with each sample solutions (1000 mL) to form coarse emulsions with high-speed shear homogenisation (Ultra-Turrax T10 homogenizer, Germany) at 15000 r/min for 60 s. Following that, the emulsions were further homogenized through a high-pressure homogenizer (SAMRO Homogenizer Co., Ltd., China) at a pressure level of 30 Mpa for 10 s, repeating this operation for two cycles. The emulsions were stored at 4 °C for further study.

### Influence of pH, temperature and ionic strength on emulsion stability

The fresh emulsions were divided into 3 groups, and each respective group sample contained 15 mL of the emulsion in a glass bottle. The emulsions in the first group were heated at different temperature including 60, 70, 80, 90 and 100 °C for 30 min. Each emulsion in the second group was respectively dealt with different pH value of 3.0, 4.0, 5.0, 6.0 and 7.0 by hydrochloric acid and sodium hydroxide solutions. The emulsions in the third group were respectively added with sodium chloride powder to reach the salt concentrations from 0.1 to 0.5 M. Lastly, the emulsions were stored at 4 °C for a night and the droplet sizes were measured by using a Malvern MasterSizer 2000 (Malvern Instruments, Worcestershire, UK). The results were reported as the surface-weighted mean diameter, d_4,3_.

### Statistical analysis

All data were presented as the mean ± standard deviation (SD) with Origin 8.0 software and were repeated in at least three independent experiments.

## Results and Discussion

### Formation of OVA-CMC complexes at pH 4.7

**Figure 1:**
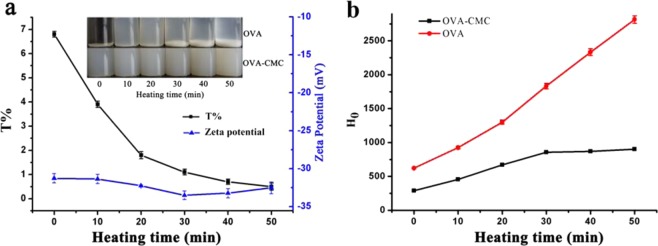
The turbidities (T%: transmittance%) and ζ-potentials of OVA-CMC with a time gradient (0, 10, 20, 30, 40 and 50 min) at 60 °C (the inset was the digital images of free OVA and OVA-CMC with heating at the same conditions) (**a**); the indexes of surface hydrophobicity (H_0_) of OVA and OVA-CMC with heating at the same conditions (**b**).

Since the isoelectric point of OVA is around pH 4.7^13^, the PDI values of OVA (0.753 ± 0.032) and OVA-CMC (0.583 ± 0.015) solutions were all above 0.5, indicating a poor dispersity with large size dimensions and heterogeneous distribution. OVA molecules tended to aggregate due to lack of surface charges. Because of the thermal instability of free OVA, significant amount of precipitates were observed at both before and after heating condition at 60 °C, pH 4.7 (inset of Fig. 1a), and dramatically more precipitates were found in heated samples. However, the existence of CMC could significantly inhibit the aggregation of OVA due to the strong electrostatic repulsions as reflected by their zeta potential values being greater than −30 mV. Our results are in accord with a previous study, which demonstrated CMC could significantly enhance the stability of OVA near its isoelectric point^14^. Furthermore, it was obvious to see that the OVA-CMC complex became opaque at all conditions, as shown in Fig. 1a, suggesting that the intermolecular interactions between OVA and CMC led to the formation of a stable colloidal system. Although there was little surface charge in OVA at its isoelectric point, the electrostatic interactions may still exist between positively charged segments in OVA and negatively charged CMC, besides the strong hydrophobic interactions^8^. Turbidities and zeta potentials of free OVA could not be determined due to the existence of the large aggregates (Fig. 1a). The transmittance (%) of OVA-CMC became smaller with increasing the heating time (Fig. 1a), indicating that more complex particles were formed with extended heating. This may be explained by thermal denaturation of OVA upon heating and the CMC coating around the partially aggregated OVA molecules may be strengthen by increased hydrophobic interactions under extended heating conditions, thus forming larger complex structure with higher turbidity^11^.

### The effects of heating on the surface hydrophobicity of OVA and OVA-CMC complexes

Heating would allow protein unfolding and exposition of hydrophobic residues, which was beneficial for the increasing of interfacial activity^15,16^. H_0_ value of OVA was decreased after binding with CMC (Fig. 1b) because of the coverage of hydrophilic macromolecules on the surface of the protein, resulting in a masking effect on some hydrophobic groups on the surface^17^. After heating, part of hydrophobic groups were exposed to the surface of the protein molecules, inducing the increase of H_0_ value^18^. The structure of OVA-CMC complex might become stable after heating for 30 min, as the H_0_ value reached the maximum. These results showed that heating induced the exposure of a larger number of hydrophobic groups to the surface of OVA, which may be a good indicator for better emulsifying property^10^.

### Intrinsic fluorescence of OVA binding with CMC

**Figure 2:**
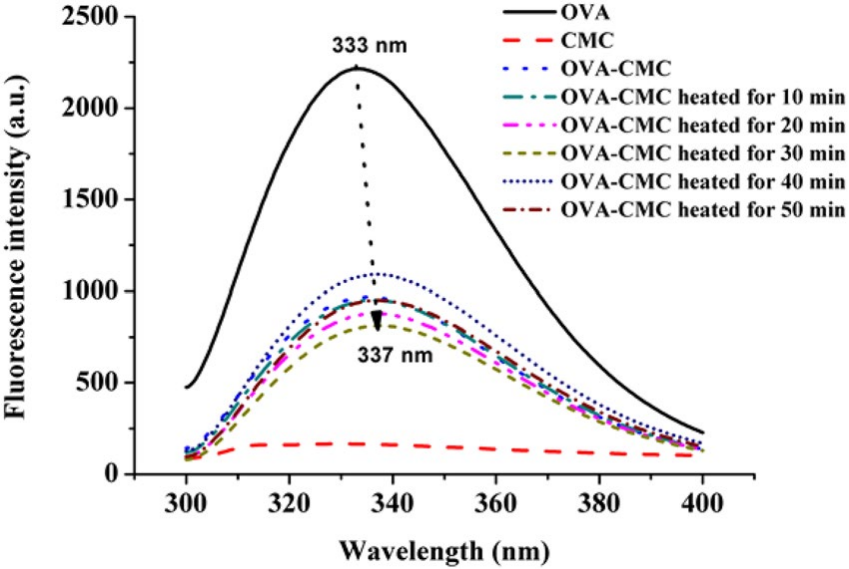
The typical intrinsic fluorescence emission profiles of OVA and heated OVA-CMC with a time gradient (0, 10, 20, 30, 40 and 50 min) at 60 °C.

The changes of intrinsic fluorescence of proteins often mirror the spatial changes of protein molecules and the interactions between proteins and other biopolymers^19^. The intrinsic fluorescence emission of proteins mainly comes from their Trp residues at the excitation wavelength of 280 nm^20^. There was obvious fluorescence quenching after heating the OVA-CMC complexes (Fig. 2). Upon heating, the protein aggregation driven by hydrophobic interaction results in the “masking” of some Trp residues and thus significantly lowers the quantum yield of fluorescence^21^. The molecular rearrangement often occurs in the protein-polysaccharide complex induced by the hydrophobic interaction^22^. The fluorescence intensity reached minimum when heated for 30 min, indicating the largest thermal denaturation. This result was in good agreement with the H_0_ analysis. After heating for 30 min, OVA achieved a moderate denaturation. Then, the secondary structure of OVA unfolded and Trp residues became to expose with further heating and fluorescence intensity increased slightly. Moreover, it is noteworthy that a distinct red-shift (about 4 nm) was observed (shifting from 333 nm to 337 nm) in the heated complex spectra, which indicated that the Trp residues of OVA were transferred from the endogenous hydrophobic region to the surface and they were exposed to a more hydrophilic microenvironment^21^. This is likely a result of the stronger hydrophobic interactions between CMC and OVA upon extended heating.

### Secondary structure changes of OVA upon interacting with CMC

**Figure 3:**
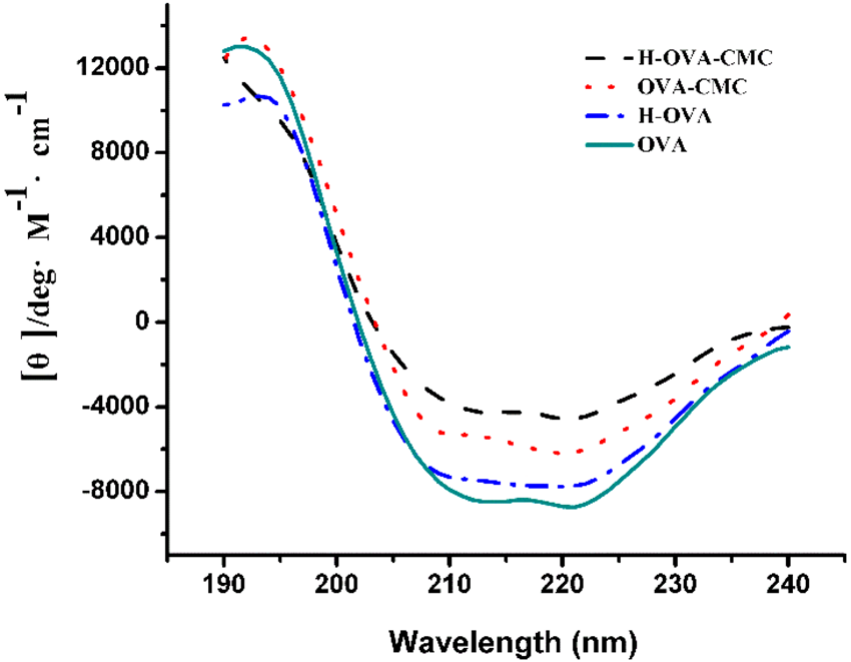
Far-UV CD spectra of OVA, OVA-CMC and H-OVA-CMC samples.

Far ultraviolet CD measurement is usually used to analyze the changes of the secondary structure of proteins which are caused by combining with other polymers^23,24^. As shown in Fig. 3, the two negative minima around 209 and 222 nm in CD spectrum of OVA revealed the existence of polypeptide chains in α-helical conformation. The band at 209 nm corresponds to π-π* transition of the α-helix while the band at 222 nm is assigned to π-π* transition, for both the α-helix and random coil^25^. The ellipticity at 208 nm is often used as a standard measure of helical content of proteins^26^. It was observed that there was a decrease of ellipticity magnitude at the negative band from 208 to 220 nm in H-OVA (Fig. 3), which indicated the reduction of α-helix structure and a partial protein unfolding. That is to say, the thermal denaturation of OVA was indeed induced under the studied heating condition^27^. Furthermore, a greater reduction of ellipticity magnitude was observed in unheated OVA-CMC sample, as well as further weakening effect upon heating. This observation revealed that the binding between CMC unfolded OVA had a significant impact on the spatial structure of OVA and further improved its thermal stability.

### FTIR study

**Figure 4:**
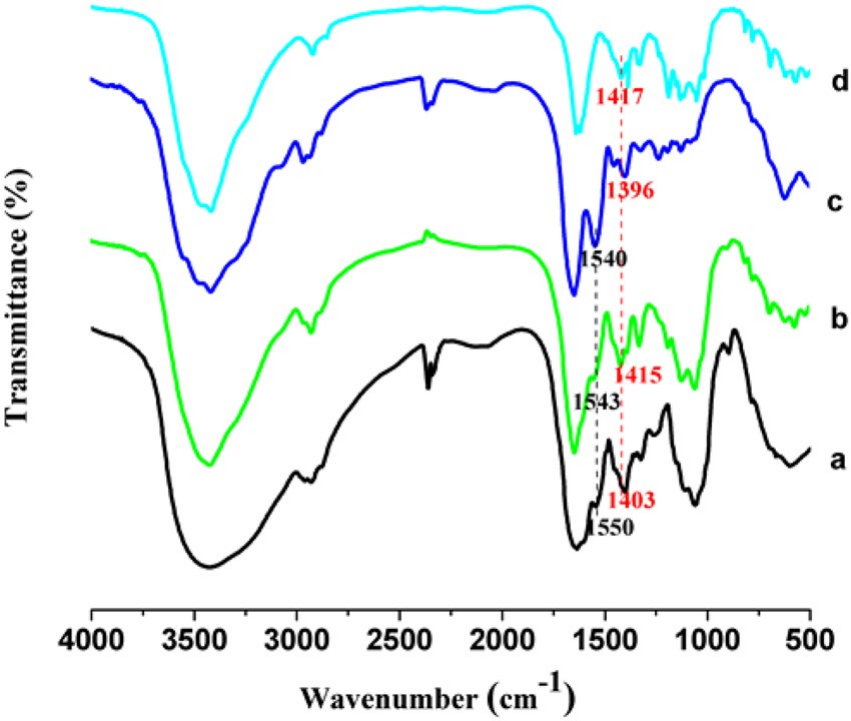
The FTIR spectra of OVA-CMC (**a**); H-OVA-CMC (**b**); OVA (**c**) and CMC (**d**).

The spectra of OVA, CMC, OVA-CMC and H-OVA-CMC are shown in Fig. 4. In the spectra of CMC, the peaks at 1417 cm^−1^ were due to the symmetric stretching vibrations of carboxyl groups^20^. The peak of the vibration of the secondary NH bonds is at 1531 cm^−1^ ^28^, and the peaks of 1540 cm^−1^ are attributed to C=O bonds^11^. In the spectra of OVA-CMC and H-OVA-CMC, the symmetrical stretching vibrations of the carboxyl groups of CMC were shifted from 1417 cm^−1^ (free CMC) to 1415 cm^−1^ and 1403 cm^−1^, respectively. The peaks of the secondary NH bending of OVA in OVA-CMC and H-OVA-CMC were shifted from 1540 cm^−1^ to 1550 cm^−1^ and 1543 cm^−1^.The changes in the peaks of C=O bonds and the secondary NH bending confirmed the spatial changes of OVA protein structure, resulting in the exposure of more hydrophobic clusters and increase the hydrophobicity interaction between OVA and CMC.

### Impact of heat treatment, pH and ionic strength on the stability of emulsion

**Figure 5:**
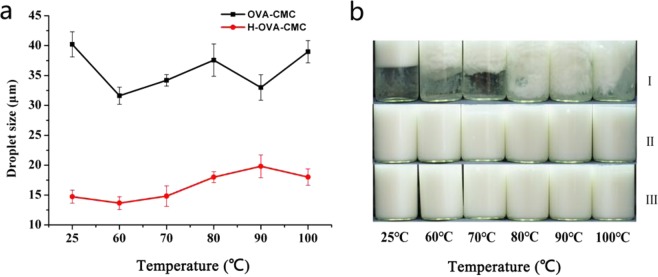
Mean droplet sizes of the emulsions prepared by OVA-CMC and H-OVA-CMC (**a**) and the digital pictures of the emulsions prepared by OVA [I], OVA-CMC [II] and H-OVA-CMC [III] (**b**) under heating at 60 °C, 70 °C, 80 °C, 90 °C and 100 °C for 30 min.

In the food industry, thermal treatment and acidification are commonly applied to ensure the shelf-stability of beverage emulsions^29^. In our study, the droplet sizes of thermal treated emulsions were measured and presented in Fig. 5a. Notably, significant flocculation was observed in the emulsion sample prepared with OVA alone (sample I in Fig. 5b), which indicated the thermal instability and demulsification effect. Unlike that, the emulsions prepared by OVA-CMC and H-OVA-CMC showed no creaming phenomenon across all heating temperatures, and the droplet sizes of these emulsions remained similar after heating at different temperatures, providing strong evidence of their superior thermal stability. The surface of CMC molecules with a large number of highly negatively charged carboxylic groups provided the strong electrostatic repulsion among the emulsion droplets. Furthermore, the droplet size of the emulsions prepared by H-OVA-CMC was significantly smaller than that prepared by OVA-CMC. As is well known, the smaller droplet size represents the better emulsifying property due to the exposure of more surface hydrophobic moieties of OVA after heating^30^.

**Figure 6:**
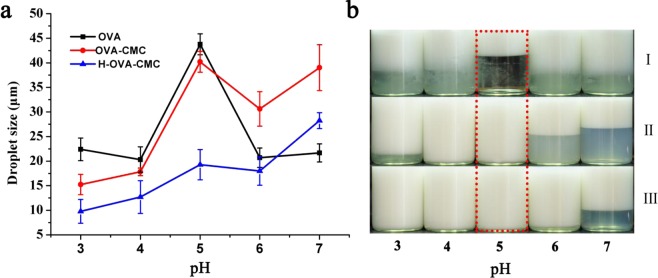
Mean droplet sizes of the emulsions prepared by OVA, OVA-CMC and H-OVA-CMC (**a**) and the digital pictures of the emulsions prepared by OVA [I], OVA-CMC [II] and H-OVA-CMC [III] (**b**) after adjusting the pH values of 3.0, 4.0, 5.0, 6.0 and 7.0.

The emulsions stability was further assessed under various pH conditions and the results are shown in Fig. 6. The largest droplet size (Fig. 6a) and the most significant creaming effect were observed (Fig. 6b) in free OVA prepared emulsion at pH 5. This is conceivable because of the weak electrostatic repulsions near the isoelectric point of protein resulting in the aggregation of emulsion drops^31^. OVA-CMC and H-OVA-CMC prepared emulsions exhibited significantly better stability against creaming with the variation of pH values (Fig. 6b), owning to the higher net surface charges of the emulsion droplets by the existence of negatively charged CMC molecules. In comparison, the droplet sizes of H-OVA-CMC prepared emulsions exhibited the best stability across all pH conditions. The strong hydrophobic interaction between OVA and CMC in H-OVA-CMC made the complex structure to be more compact, which was beneficial to the emulsion stability against the alteration of pH.

**Figure 7:**
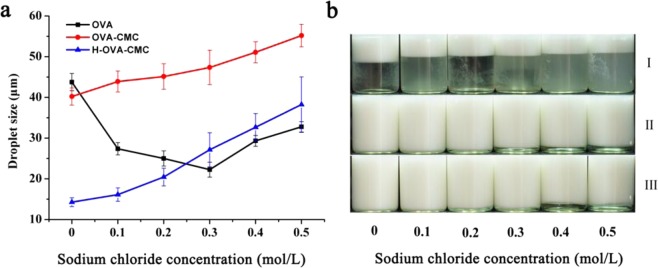
Mean droplet sizes of the emulsions prepared by OVA, OVA-CMC and H-OVA-CMC (**a**) and the digital pictures of the emulsions prepared by OVA [I], OVA-CMC [II] and H-OVA-CMC [III] (**b**) with the sodium chloride concentrations of 0, 0.1, 0.2, 0.3, 0.4 and 0.5 M.

The droplet sizes of the emulsions with the effect of Na^+^ concentrations are shown in Fig. 7a. Different types of electrostatic interactions, such as intermolecular, intramolecular and particle interactions, are all susceptible to strong ionic strength, due to the screening effects of salt ions on the colloidal electrolytes^12^. High salt concentrations could easily screen the surface charges of the polymers or complexes, making them lose their emulsification and stabilization capabilities^25^. Lack of sufficient electrostatic repulsion, creaming appeared in the emulsion prepared by free OVA near its isoelectric point (Fig. 7b). By comparison, CMC could significantly inhibit the creaming of the emulsions, although the droplet sizes of the emulsions prepared with both OVA-CMC and H-OVA-CMC became larger with increasing salt concentrations. Moreover, slight flocculation was observed in the emulsions prepared with both OVA-CMC and H-OVA-CMC, which resulted in the increase of the droplet sizes^32^. As shown in Fig. 7b, the emulsion prepared by OVA-CMC appeared a little creaming at Na^+^ concentration above 0.3 M, while the creaming was not observed in the emulsion prepared by H-OVA-CMC until reaching Na^+^ concentration 0.4 M and above. The exposure of hydrophobic clusters of OVA after heating enhanced the hydrophobic interaction between OVA and CMC, leading to the better stability of the emulsions under high ionic strength conditions.

**Figure 8:**
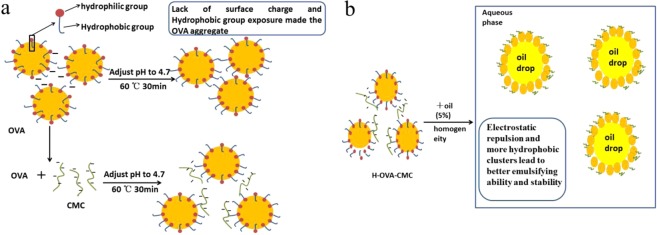
Schematic diagram of the preparation of H-OVA-CMC (**a**) and H-OVA-CMC prepared emulsion (**b**).

The schematic diagram of H-OVA-CMC stabilized emulsion was shown in Fig. 8. OVA was easily aggregated at pH 4.7 due lack of surface charges, and heating treatment further induced the exposure of hydrophobic groups leading to sedimentation (Fig. 8a, upper panel). Nevertheless, complexation with negatively charged CMC significantly inhibited the OVA aggregation via intermolecular electrostatic repulsions (Fig. 8a, lower panel). Moderate heating of the OVA-CMC complex at 60 °C for 30 min (H-OVA-CMC) further not only maintained the exposure of hydrophobic clusters of OVA, but also stabilized the complex structure via electrostatic and steric repulsions (Fig. 8b). Collectively, H-OVA-CMC demonstrated the significantly improved the emulsifying property and stabilization effect on the emulsions, comparing with free OVA and unheated OVA-CMC complex.

## Conclusions

In this study, OVA and CMC were mixed at pH 4.7 with heating at 60 °C for 10 to 50 min. The results of turbidities, zeta potentials and intrinsic fluorescence spectra revealed that weak electrostatic and strong hydrophobic interactions co-existed between OVA and CMC. CMC dramatically inhibited the thermal aggregation of OVA due to its strong electrostatic repulsion. Following that, CD and FTIR spectra were used to further characterize the binding property of heated OVA-CMC under moderate heating condition at 60 °C for 30 min (H-OVA-CMC). It was revealed that heating induced thermal denaturation and exposure of more hydrophobic clusters of OVA molecules, which not only increased the hydrophobic interactions between OVA and CMC, but also introduced steric repulsions, significantly improving the colloidal stability. The emulsion prepared by H-OVA-CMC showed excellent emulsifying ability and stability against heating, pH changes and ion strength, compared with free OVA and unheated OVA-CMC complex. Our research provides a new insight for application of OVA-CMC complex in acidic protein and emulsion beverages.
